# The Ascent of Miniproteins

**DOI:** 10.1021/acs.biochem.6c00311

**Published:** 2026-06-18

**Authors:** Yejong Yoo, Vikas Nanda

**Affiliations:** Department of Biochemistry and Molecular Biology, Robert Wood Johnson Medical School and the Center for Advanced Biotechnology and Medicine, 12287 Rutgers University, Piscataway, New Jersey 08854, United States

## Abstract

Miniproteins are present across all branches of life and play central roles in diverse biological processes, but their existence fundamentally challenges our assumptions about the minimal sequence, structural, and energetic requirements necessary for protein stability and function. More than molecular curiosities, miniproteins may offer a window into the earliest catalysts that emerged at the origin of life. Here, we focus on metal-containing miniproteins, where coordination chemistry can impart both structural stability and redox activity essential for metabolism. We ask whether primordial peptides could have evolved into complex proteins through miniprotein intermediates by surveying natural miniproteins as snapshots along an evolutionary trajectory, and examining engineered miniproteins that can model the gaps between these snapshots. Finally, we explore whether miniproteins continue to evolve today—either recapitulating early evolutionary processes or giving rise to entirely new folds and functions.

## Graphical abstract

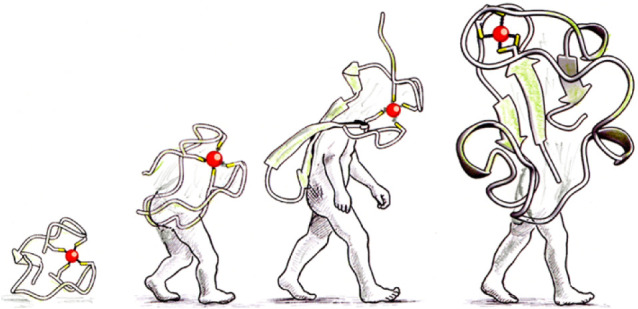

The origin of life on Earth was a phase transition from thermodynamically driven chemical cycles to biologically regulated reaction networks. A key component of this transition was the emergence and elaboration of information-containing polymers—proteins and nucleic acids. The result of this process is modern genomes stretching from hundreds of kilobase to hundreds of gigabases wherein proteins are encoded with lengths up to tens of thousands of amino acids. Molecules of this size and complexity can only exist in living systems where fidelity is maintained through genetic mechanisms. In contrast, the first molecules were likely much shorter, given that the number of potential sequence combinations increases dramatically with length. If we were to generously assume the average peptide has a solubility of 1 millimolar, then ∼6 × 10^5^ unique sequences would fit into a 1 femtoliter volume, the size of a bacterial cell. For a 4 aa peptide, the number of combinations would be 20^4^ or 1.6 × 10^5^ unique sequences, leaving little room for much else in an early cell. Even in the entire volume of the Archean ocean (∼10^21^ liters), one could only fit a few copies of each sequence for a 30 aa peptide. Clearly, if a prebiotic cycle incorporated protein polymers, it would not easily have depended on an enumerative stochastic polymerization.

Ancient peptides likely occupied regions of sequence space that chemistry could actually visit: short, compositionally biased, and highly conditioned by the environment in which they were formed. Systems chemistry approaches demonstrate that peptide space can be navigated under thermodynamic control rather than by uniform combinatorics. In the laboratory, dynamic peptide libraries undergo cycles of condensation and hydrolysis, shaped by ligand binding and self-assembly, driving the product distribution toward a subset of favored sequences.^1,2^ In more prebiotic-Earth-relevant models, such as oscillating water activity to simulate wet–dry cycles in surface ponds, thermodynamically driven reduction in combinatorial complexity is observed.^3^ Chemical autocatalytic cycles driven by peptide–peptide molecular recognition and templating can also reduce complexity and amplify the synthesis of specific sequences.^4–6^ In this view, perhaps the earliest peptides did not explore sequence space uniformly but were steered by thermodynamics and chemistry toward a restricted set of sequences amenable to evolutionary elaboration.

Then, how small can a peptide be and still matter to evolution? Evolution selects for fitness, which, in turn, depends on how molecular structure modulates function. Short peptides are generally not well-structured in the classic sense of readily folding into a unique, native state. Rather, they are highly dynamic and disordered, or they occupy broad conformational ensembles. Short peptides statistically disfavor regular secondary structure due to the low probability of nucleation events in helix–coil transitions, and the hydrogen bonds that enforce such structures are only marginally stable in water.^7,8^ Additionally, short peptides lack the ability to shield hydrophobic amino acids from water, removing one of the core stabilizing processes that promote structures in larger proteins.^9^ Thus, there is a conundrum—as peptides get longer, their capacity to fold increases, but so does the combinatorial complexity of sequence space. What is the minimal length required to fold?

Folding is governed by the balance of enthalpic and entropic processes that stabilize the native state versus those that stabilize the disordered ensemble. Conformational entropy stabilizes the unfolded state, with estimates of ΔS upon folding ∼1–2 kcal/mol·residue.^10,11^ The burial of a side-chain methyl group upon folding stabilizes the folded state by about the same amount,^12^ meaning that, on the whole, one sidechain methyl group can restrict the conformation of one amino acid. Based on this logic, a fully buried leucine could stabilize itself and up to four additional residues. Assuming an idealized spherical protein topology, the number of buried positions (*n*_*b*_) scales with length (*n*) as *n* ^1/3^ – *n*_*b*_^1/3^ = *k*, where *k* is a parameter related to the thickness of the boundary between the core and the protein surface.^13^ This scaling can be used to estimate ΔG of folding based on length (Figure 1). At very short lengths (*n* < 10 aa), ΔG increases near-linearly with length, as the polypeptide cannot bury a hydrophobic core to compensate for increasing chain degrees of freedom. Assuming all buried residues are leucine-sized, hydrophobic interactions begin to overtake entropy around *n* = 20 aa, with folding being favorable (ΔG < 0) at lengths of *n* = 34 to 54 aa, depending on the thickness parameter, *k*. Of course, this model is a drastic oversimplification, both in terms of protein topology and folding energetics; proteins are not spheres, and folding is stabilized by more than just hydrophobic groups and destabilized by more than just chain entropy. The model serves as a framework for exploring the relationship between length and stability for monomeric, soluble folds.

**Figure 1:**
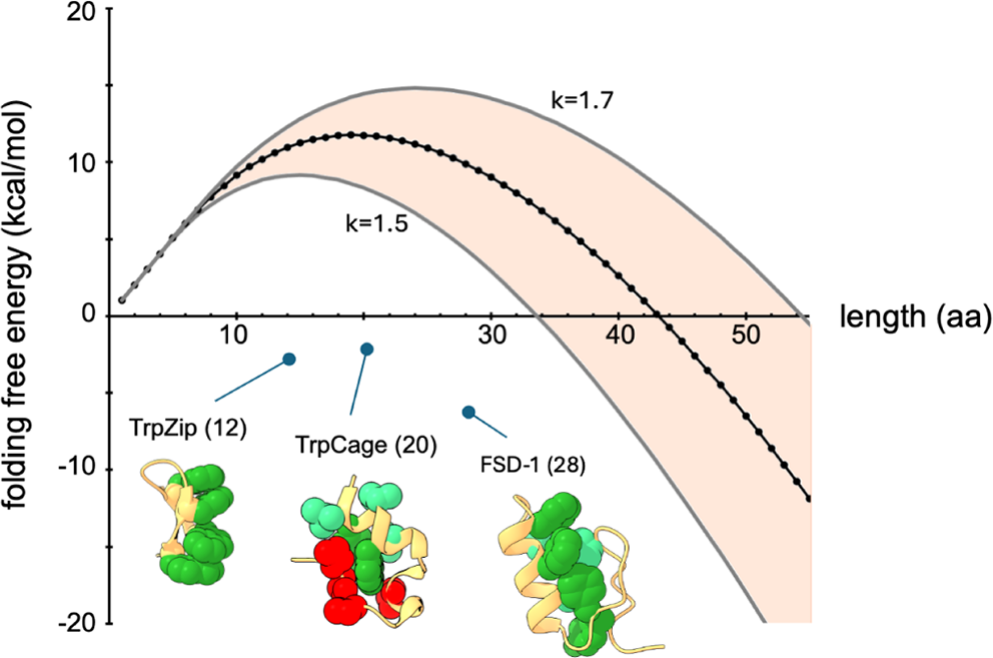
Estimating folding constraints on protein length. The number of buried positions (*n_b_*) in an idealized, spherical protein scales with total sequence length (*n*) as *n* ^1/3^ – *n_b_* ^1/3^ = *k*; where *k* = 1.62 ± 0.13.^13^ Δ*G_folding_* versus length can be approximated as the entropic cost of fixing an amino acid backbone in the native conformation (3.4 cal/K•mol•position, 310 K^10^) opposed by the gain of burying hydrophobic groups (1.1 kcal/mol per −CH2– group^12^). Assuming all buried positions are leucine, a protein must be at least 43 residues long and bury seven positions in order to fold. This estimate varies based on the thickness *k* of the solvent-exposed layer, giving a range of minimal sizes from 34 to 54 residues. Notable exceptions to this size limit have been designed—e.g., TrpZip,^14^ TrpCage,^15^ FSD-1.^16^ In these designs, favorable folding was engineered by optimizing core hydrophobicity (atom coloring: dark green—aromatic groups, light green—aliphatic groups) and/or reducing backbone entropy (red—proline).

While the proposed model places a lower bound of n ∼34 on miniproteins stabilized by a hydrophobic core, many designed proteins are, in fact, much shorter. FSD-1, modeled from a 28-aa zinc-finger domain, was computationally redesigned, replacing the metal site with an extensive hydrophobic core composed mostly of phenylalanine.^16^ The large side chain of phenylalanine provides an extensive hydrophobic surface for folding, supplemented by aromatic stacking forces.^17^ The large side chain also reduces backbone and side-chain entropy in the unfolded state.^18^ The Trp-cage, developed from exendin-4, a natural peptide hormone discovered in the saliva of the Gila monster lizard, is only 20 aa, and shows cooperative, two-state folding.^15^ The hydrophobic core is minimal, with a central tryptophan packing against tyrosine and isoleucine, again taking advantage of size and aromaticity. Several prolines likely also stabilize the native state by disfavoring unfolded-state conformational flexibility. One of the smallest designed miniproteins may be the TrpZip, coming in at a meager 12 aa, with a pseudocore on one face of a β-hairpin comprising four tryptophans.^14^ The amphiphilic character of the indole group on tryptophan may support the assembly of this exposed core while maintaining solubility—akin to the unique role of tryptophan in membrane proteins at the water–lipid interface.^19^ High-throughput design and characterization of large-scale synthetic miniprotein libraries (40–43 aa) establish that successful designs optimize hydrophobicity along with other features: secondary-structure propensities, helix capping, and electrostatic interactions.^20^ These and many other engineered proteins are triumphs of protein design, taking a maximalist approach to optimizing stability within a minimal scaffold.^21,22^

Do natural miniproteins match designed ones, folding within a small footprint by optimizing the core through aromatic interactions, incorporating rigid prolines, or maximizing electrostatics? Examples such as the exendin-4 hormone that led to the Trp-cage do exist, but they seem to be the exception rather than the norm. A survey of 150 sequences from the *E. coli* small proteome (verified gene products <50 aa)^23^ found that very few had annotated functions, and none had reported experimental structures. If we use the model of core hydrophobicity versus chain flexibility, sequences of the *E. coli* small proteome largely fall on or under the scaling relationship for *k* = 1.62, assuming core residues are leucine (Figure 2). This suggests that many of these peptides and miniproteins maximize hydrophobic content by favoring larger residues, including abundant aromatics. Alternatively, the boundary thickness *k* may be effectively smaller for peptides and miniproteins due to the breakdown of the sphere approximation at these length scales. A more appropriate way to consider these small proteins is to look at their actual structures.

**Figure 2:**
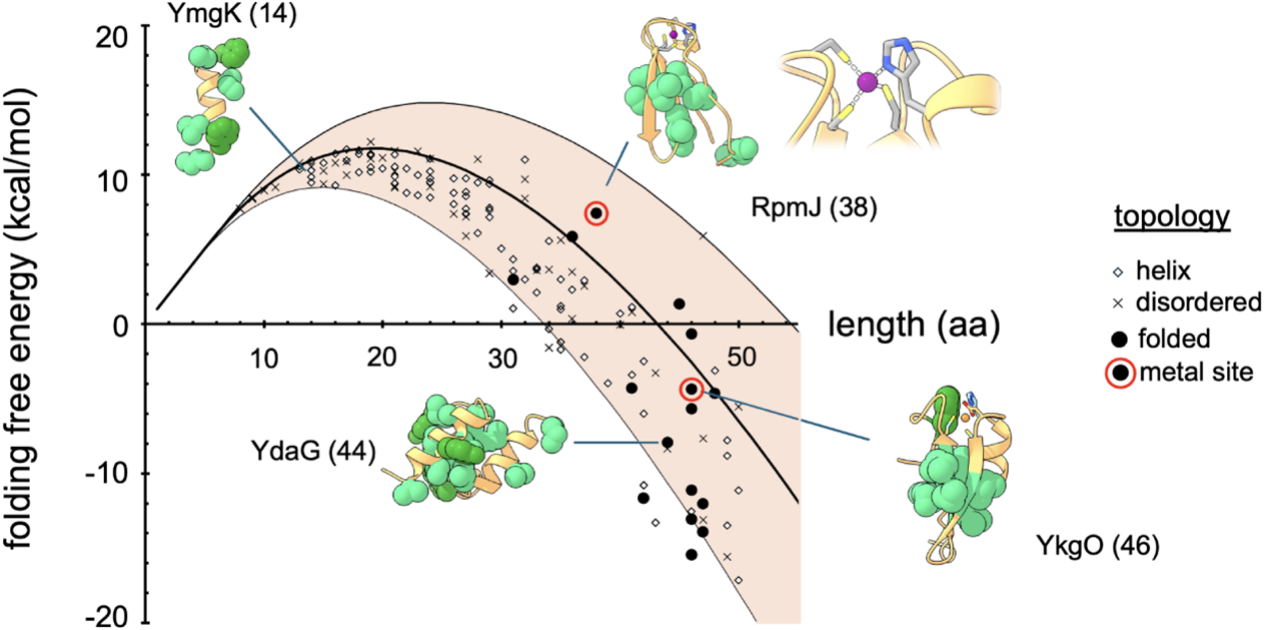
Estimated*E. coli*peptide and miniprotein folding. An estimated ΔG of folding was calculated using the spherical approximation of the buried fraction of amino acids vs length^13^ for 150 putative miniproteins from *E. coli* (size <50 aa).^23^ The line shows the ideal value for *k* = 1.62. AlphaFold-2 and ESM-2 structural models^24,25^ of peptides below 35 amino acids were all hydrophobic single α-helices (e.g., YmgK) or disordered. Some miniproteins of size >35 aa with extensive hydrophobic cores (YdaG) are predicted to fold. Others, such as RpmJ, have ΔG > 0 but may use metal coordination to stabilize the fold (modeled here with a Zn^2+^ ion). YkgO has both a hydrophobic core sufficient for folding and a putative metal-binding site (here modeled with Cu^2+^). (atom coloring: dark green—aromatic groups, light green—aliphatic groups). Folded topologies were identified as Alphafold-2 models with maximum contact orders >9 residues. Model topologies were single-helix if the fraction of residues classified as helical by DSSP was >0.6.^26^ Otherwise, they were defined as disordered.

Unfortunately, very few experimental structures exist for the *E. coli* small proteome, leaving us to consider models predicted by AlphaFold-2 ^25^ and ESM-2.^24^ No sequences smaller than *n* = 31 aa in the *E. coli* small proteome are predicted to form compact tertiary folds. Instead, they are modeled as single helices, often very hydrophobic, suggesting they oligomerize, interact with other proteins, or associate with membranes. Others are largely disordered. While these models are informative, it is important to note that AlphaFold and ESM do not always reliably model shorter sequences.^27^ Several of the longer miniproteins are predicted to adopt tertiary folds, such as YdaG, with a three-helix topology. The three-helix fold was the most designable scaffold in high-throughput miniprotein libraries,^20^ suggesting it may be an evolvable topology for biological selection. Many of the natural miniproteins in this data set are consistent with the scaling relationship for length vs folding, where sequences below the *n* = 34 aa threshold do not seem to form folded structures. These short peptides may be functional and subject to evolutionary selection but not in the context of soluble, monomeric folds.

An interesting exception to the length-folding scaling relationship is the *E. coli* miniprotein RpmJ, which lacks a sufficient hydrophobic core to fold (Figure 2). RpmJ associates with the large ribosomal subunit (designated as bL36 in this context), and its basic surface likely facilitates strong electrostatic interactions with the rRNA. Both AlphaFold-2 and ESM-2 models of RpmJ included a pair of loops with putative first-shell metal ligands (Cys, His), and AlphaFold-3 models predicted a Zn^2+^ ion coordinated at this site. Knockout of this protein alters zinc homeostasis in the cell,^28^ and RpmJ homologues in experimental ribosome structures confirm zinc binding at this site.^29^ A few other examples of putative metal-binding folds are found in this set, such as YkgO, a protein of unknown function that may bind metals through a Cys-Cys-His-Asp site. Metal binding can provide a massive boost to stability while also clamping down on chain entropy. Measurements of Zn^2+^ binding to short disordered peptides (16 aa) showed that metal coordination can provide up to 22 kcal/mol of stabilization.^30,31^ The incorporation of metal-coordinating ligands into a peptide sequence could provide an evolutionary path from small metallopeptides <35 aa to metallo-miniproteins able to support both metal sites and hydrophobic cores.

Metals not only confer stability and constrain structure, but for transition metals, the Lewis acidity of low-lying orbitals allows for reactivity and catalysis.^32^ As such, metallopeptides are a fertile starting point for evolutionary selection in an origin-of-life scenario.^33^ Metal coordination likely provides thermodynamic constraints on sequence space in prebiotic polymerization scenarios, stabilizing select sequences against degradation.^34^ There are cases of small metallopeptides in nature, such as the antimicrobial bacitracin, where coordination of zinc and sodium stabilizes an active, amphipathic conformation of the peptide^35^ (Figure 3). Most examples of peptides that bind metals and show redox or catalytic activity come from synthetic molecular designs. For example, the helical peptide Cy-AA-EK (17 aa) dimerizes around a heme and is stabilized by hydrophobic interactions with the porphyrin, disulfides, and metal–ligand bonds.^36^ The 11 aa METP binds as a dimer to iron or zinc and can undergo several oxidation/reduction cycles before losing activity.^37^ Two isoleucines, one from each chain, form a minimal hydrophobic core. Ambidoxin is 12 aa long and binds a 4Fe-4S cubane cluster. It is stabilized by both metal coordination and the inclusion of D-amino acids to reduce the conformational flexibility of the unfolded, apo form.^38^ The stability of the fold confers high activity, with ambidoxin able to cycle over a thousand times between oxidized and reduced states. Nickelback, a dinuclear nickel peptide 13 aa long, was demonstrated to be capable of electrocatalytic evolution of H_2_.^39^ Similar chemistries have been achieved with tripeptides: Gly-Gly-His can bind cobalt and electrocatalytically evolve H_2_,^40^ and Cys-Gly-Cys mimics the active site of acetyl-CoA synthase.^41^ Short, cysteine-containing metallopeptides can generate electrochemical gradients across membranes.^42^ Laboratory models suggest that metal coordination enabled short peptides to achieve functional complexity far beyond their length, positioning them as compelling candidates for early, selectable catalysts in prebiotic and protobiological environments.

**Figure 3:**
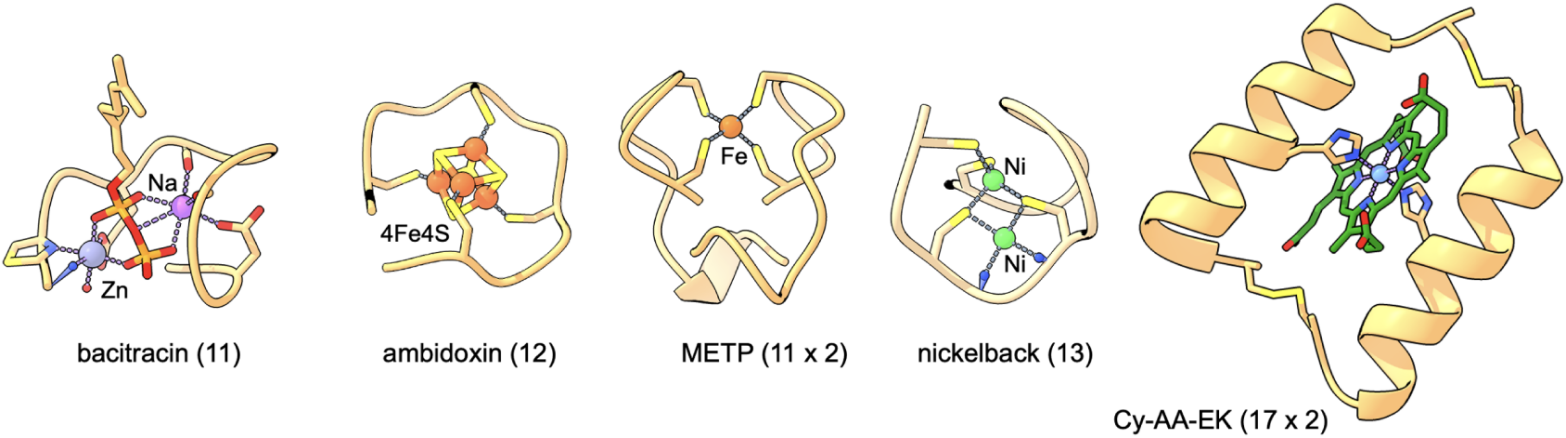
Metal coordination stabilization of short peptides <20 aa. Bacitracin^35^—PDB ID 4k7t s; ambidoxin^38^—molecular model; METP^37^—AlphaFold-3 model;^43^ nickelback^39^—molecular model; Cy-AA-EK^36^—PDB ID 1pbz. Only residues involved in first-shell metal coordination are highlighted.

Another route by which very short peptides may achieve stable, well-defined structures is through disulfide bonding. Multiple disulfides are a common strategy in naturally occurring peptide toxins and hormones, including conotoxins, scorpion and spider venoms, and hepcidin, where densely cross-linked cysteine networks stabilize compact folds in sequences as short as 10–30 residues. In contrast to hydrophobic cores, which require sufficient chain length to exclude solvent, disulfide bonds constrain the unfolded ensemble directly by covalently linking distant segments of the chain, thereby reducing conformational entropy. A single disulfide bond may contribute ∼2–5 kcal/mol to short peptides where entropy reduction dominates.^44^ In disulfide-rich miniproteins, the cumulative stabilization enables well-defined tertiary structures in the absence of extensive hydrophobic packing. An extreme example is the sea squirt vanabin, where arrays of disulfides along a helix–helix interface replace a traditional heptad pattern of hydrophobic amino acids.^45^ From an origin-of-life perspective, disulfide formation offers an alternative route to structural preorganization that does not rely on longer chains with hydrophobic cores. However, in the weakly reducing environment of the early Earth, free thiols would have been energetically favored over disulfides. Peptides and miniproteins stabilized by disulfides would either have required more oxidizing microenvironments, perhaps driven by abiotic photochemistry,^46,47^ or tuning of the disulfide energetics by second-shell interactions contributed by the peptide.^48^

Metal-stabilized miniproteins are unlikely to have emerged in isolation. Other origin-of-life frameworks emphasize RNA and ribozyme chemistry, mineral surfaces and condensates as concentrative and templating environments, protometabolic reaction networks catalyzed by metals and small molecules, and heterogeneous noncoded polymers or mixed peptide–nucleotide assemblies.^49–52^ We therefore treat metallopeptides and metallominiproteins as one plausible branch within a pluralistic prebiotic landscape, particularly compelling because metal coordination can couple stabilization and catalysis in molecules still far smaller than most modern proteins.

One could infer from these natural and design case studies that size matters little to evolution and that everything from tripeptides to full proteins is amenable to selection by Darwinian feedback processes. If so, it is further tantalizing to hypothesize a continuous pathway may exist, linking short metallopeptides, metal-binding miniproteins, and large metalloenzymes through evolutionary lineage.^38,53–57^ However, there is currently no direct phylogenetic evidence to support this. Many of the shortest natural metallopeptides, such as siderophores, are synthesized nonribosomally and include chemical modifications beyond the 20 genetically encoded amino acids.^58^ In such cases, selection would operate at the level of recombination of the synthetase complexes,^59^ rather than the coding sequence of the peptide itself, decoupling them from genetic elaboration. Furthermore, the peptides, miniproteins, and proteins in natural proteomes have discontinuities in sequence, structure, and function. If one exists, what would a plausible pathway across length regimes look like?

One example of a fold that spans peptides to proteins is that of rubredoxin (Figure 4). Rubredoxins are protein electron carriers involved in microbial fatty-acid hydroxylation, moving electrons from NADH-dependent reductase to the hydroxylase.^60^ Rubredoxin can be considered a miniprotein, 50–60 aa long, with a single iron coordinated by four cysteines. It inspired the design of the METP dimeric peptide, which took advantage of the C2-symmetry of the metal-binding turns of natural rubredoxin, collapsing the extensive hydrophobic core to two isoleucines—one per chain.^61,62^ Subsequently, a redesign with a short, structured linker led to METPsc1, a 25 aa with both turns in a single chain. METPsc1 was structurally and catalytically superior to METP, despite a modest hydrophobic core.^61^ Although METPsc1 was designed from first-principles, it represents a minimal solution that has also been sampled in nature. One homologue of METPsc1 was identified in the sORFdb,^63^ annotated as a Zn-ribbon domain found in *Lactobacillus*. Structural models of this 37 aa miniprotein predict the first 25 aa adopt the same topology as METpsc1, with a C-terminal disordered 12 aa tail. A structural homologue identified using FoldSeek,^64^ also starts with a 25 aa core domain followed by an unstructured C-terminal tail. Rubredoxin-like subdomains exist within larger proteins, such as adenylate kinase.^65^ Here as well, the hydrophobic core is minimal, and the domain is potentially stabilized by tertiary interactions with the remaining protein. The homology of designed and natural miniproteins highlights the potential of using reductionist design strategies to model putative evolutionary paths, a strategy that can likely be extended to other folds, particularly where metal coordination symmetry is involved.^62^ It is important to note the examples recounted here are isolated snapshots across species and functions, and a plausible alternative explanation is the convergence of independent evolutionary paths on the same fold. Larger-scale surveys of protein fold space find the rubredoxin topology recurs frequently,^66,67^ consistent with an ancient, shared origin.

**Figure 4:**
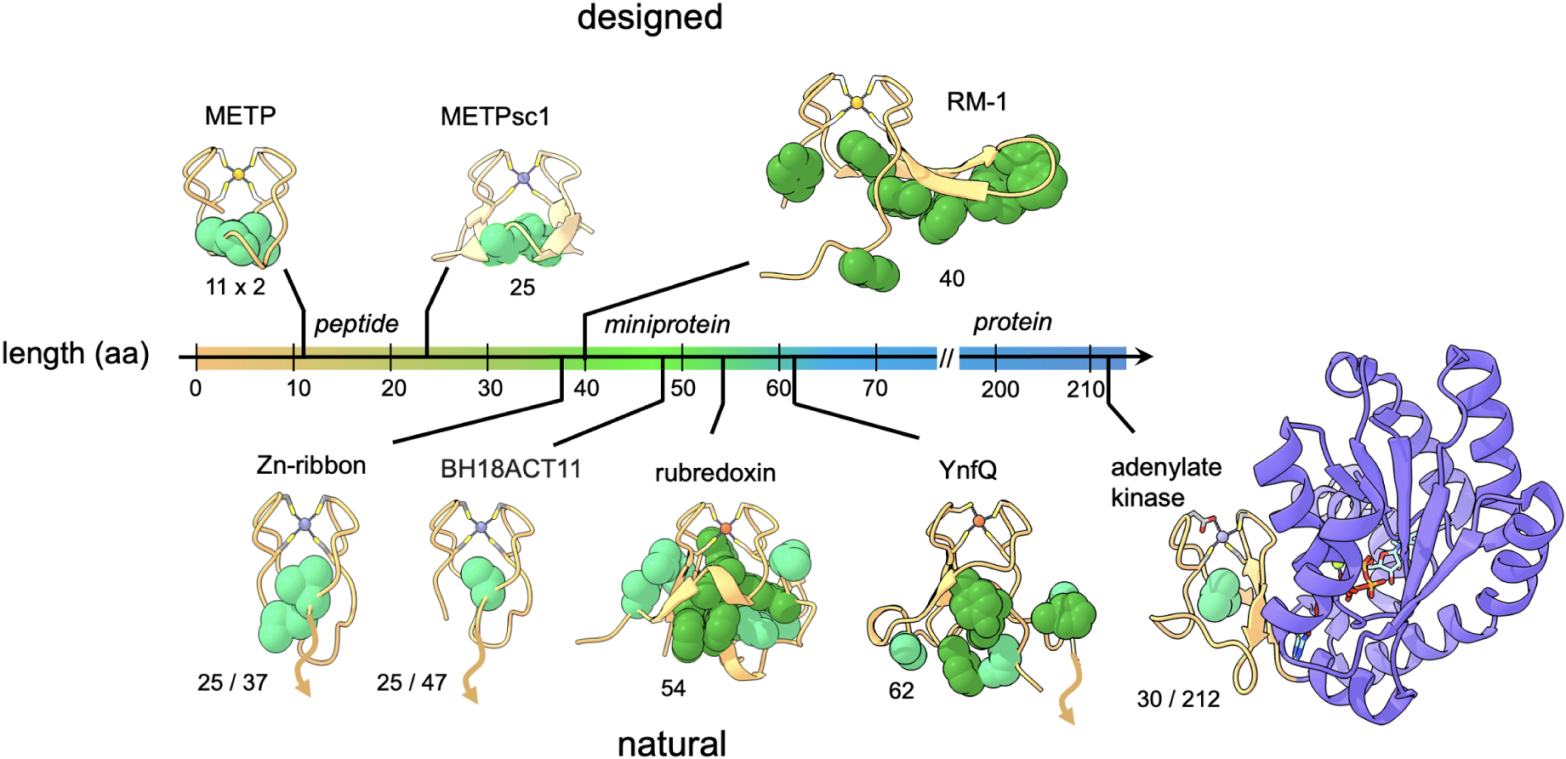
Natural and designed rubredoxin domains from peptide to protein scales. Minimal versions of the rubredoxin fold have been explored using *de novo* protein design from a short peptide dimer METP^37^ (AlphaFold-3 model bound to Fe^2+^) and the single-chain METPsc1 ^61^ (PDB ID —5sbg) to a miniprotein with an extended hydrophobic core, RM-1 ^68^ (AlphaFold-3 model bound to Fe^2+^). Natural peptides and miniproteins such as the *Lactobacillus* Zn-ribbon (GenBank—MCD5529125.1) where the first 25 of the 37 positions are similar in size and structure to METPsc1 (modeled using AlphaFold-3 bound to Zn^2+^). BH18ACT11—a hypothetical protein from an Antarctic soil metagenome—also shares this fold for the first 25 of 47 residues (GenBank—CAN5739409.1, modeled using AlphaFold-3 with Zn^2+^). Rubredoxin from the thermophile *P. furiosus* forms a compact miniprotein with an extensive hydrophobic core^69^—PDB ID—1brf. YnfQ from the *E. coli* small proteome^23^ matches the rubredoxin topology with a metal site and extensive core, modeled in AlphaFold-3 bound to Fe^2+^. Adenylate kinase from *B. subtilis* includes a small rubredoxin-like domain^65^ bound to Zn^2+^—PDB ID—1p3j. (Atom coloring: dark green—aromatic groups, light green—aliphatic groups).

The best-supported case of lineage from peptide to protein is that of bacterial ferredoxins—short 55-aa miniproteins that coordinate two 4Fe4S clusters and serve as low-potential electron shuttles between donor and acceptor enzymes in cellular metabolism, much like rubredoxins. In 1967, Margaret Dayhoff proposed that fossil evidence of ancient duplication and repetition events of much shorter peptides is buried in the sequence of modern ferredoxins.^70^ Homology between the N- and C-halves of the ferredoxin sequence and their structures clearly supports this, and ancestral sequence reconstructions suggest ancestral forms where the two halves are identical.^71^ Structural and functional continuity was demonstrated in an engineered evolutionary lineage, starting from preduplication 28-aa “semidoxin” peptides to postduplication and fusion 55-aa “symdoxin” miniproteins with symmetric N- and C-halves.^72,73^ Metal binding, thermodynamic stability, and redox energetics were preserved across this transition. The main discontinuity was in in vivo electron transfer complementation assays.^74^ The shorter semidoxins did not function well in an engineered cellular electron transfer pathway relative to the longer symdoxins, except under very low experimental oxygen levels. Consistent with this, putative semidoxin homologues in nature are limited to the genomes of anaerobic bacteria. Similarly, examples of symdoxins have also been identified where N- and C-halves are nearly identical, indicating recent duplication and fusion of semidoxins. In the very large protein regime, multiple ferredoxins are embedded in large proteins where extended chains of 4Fe4S clusters move electrons over several-nanometer distances.^75^ A discontinuity still remains at the very small scale, for peptides with lengths shorter than semidoxins (with the exception of free cysteine^76,77^ ), where ferredoxin-like 4Fe4S-binding peptides comprising only natural amino acids have yet to be identified in nature or designed.

Many other cases of peptide → miniprotein → protein lineages spawning from the origins of life likely exist. In modern fold evolution, however, trajectories are not always linear. The closest homologues of naturally occurring semidoxins are found in large, multidomain enzymes,^72^ suggesting fragmentation of a peptide from a large protein. Similarly, the rubredoxin domain of adenylate kinase is closer in structure and sequence to the 25 aa peptides, than to a 55 aa miniprotein, perhaps suggesting a fusion event that skipped the miniprotein stage entirely. What does this mean for the miniprotein proteomes present in extant life—are they all recycled fragments of larger proteins? Or does the “tape of evolution” continue to replay today,^78,79^ exploring new directions in protein space that have yet to be sampled?^80^

The question of whether some miniproteins are truly on *de novo* evolutionary trajectories or are derived from existing proteins is at the frontier of inferential methods. Phylogenetic models based on comparative analysis of nucleotide or protein sequences become increasingly unreliable as sequences diverge extensively. Structural homology is more useful for remote homology, but the structure space is sparsely determined, and many proteins have intrinsic disorder and evolve outside structural constraints. The development of protein language models (pLMs), where the high-dimensional representations of protein sequences in latent space provide information enriched by evolutionary constraints, can expose relationships that are inaccessible to tree-based phylogenetics and structure comparison.^24,81–83^

As an exploration of what insights pLMs might contribute, we examined three sets of protein sequences—bacterial miniproteins from the sORFdb,^63^ metal-binding bacterial proteins annotated as such from SwissProt^84^ and MetalPDB^85^ and bacterial proteins with no annotated metal-binding motifs. Sequences were filtered for those that included sufficient first-shell ligand amino acids (cysteine/histidine) to potentially form metal-binding sites and trimmed to 45 aa in length to equate comparisons between miniproteins and larger sequences in the other two sets. The output embeddings for all sequences were computed using the ESM-2 pLM, mapped Iatent space, and projected into clusters (Figure 5). While most miniproteins map with sequences from the other two sets, a notable fraction in the upper right of the map does not.

**Figure 5:**
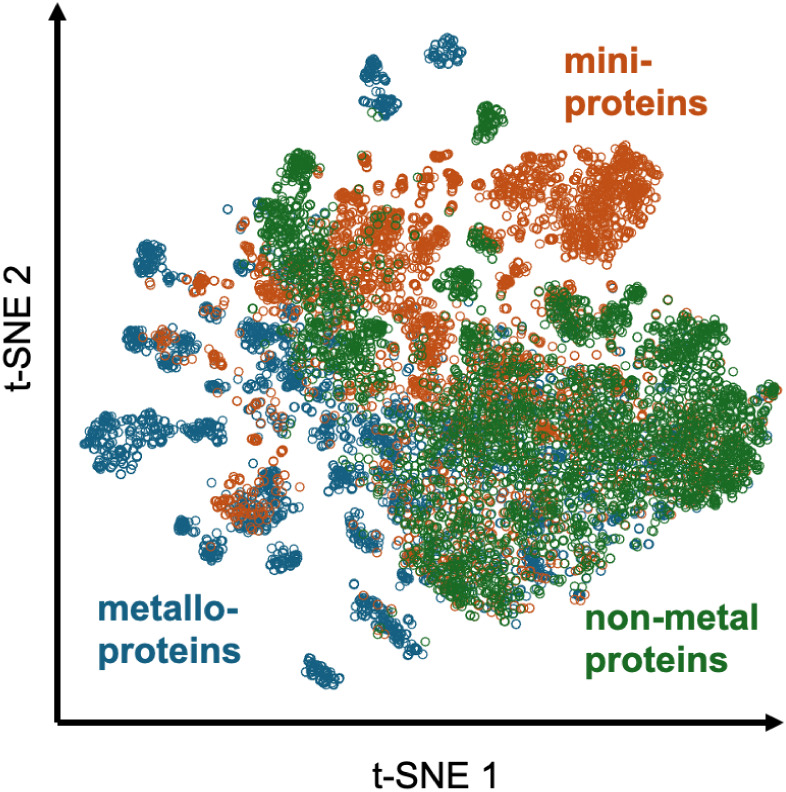
t-SNE Projection of Full-Sequence ESM Embeddings for Metalloproteins, Non-Metal Proteins, and Miniproteins. 45 aa fragments were selected from Cys/His-containing sequences to construct a metalloprotein set (source: MetalPDB^85^ + SwissProt^84^ with Fe–S annotation, 4000 sequences), a nonmetal protein set (source: SwissProt with no metal annotation, 4000 sequences), and a miniprotein set (sORFDB^63^ of length ≤ 100 aa, 3708 sequences). Output embeddings were calculated using the lightest ESM-2 model^24^ (8 M parameters, 6 layers) and reduced to two components using t-distributed Stochastic Neighbor Embedding (t-SNE).^86^ A large fraction of miniproteins cluster separately from the central manifold, apart from metallo- and nonmetal proteins.

Only a small fraction (2/150) of the *E. coli* small proteome had folds compatible with metal binding. Does this extend to a broader sampling of miniproteins? While the pLM embeddings provide a powerful way to compare sequences across evolutionary scales, t-SNE projections are designed for visualization of local neighborhood structure rather than for quantitative interpretation of global distances, and relationships inferred from t-SNE are most meaningful at the level of the nearest-neighbor composition. With this limitation in mind, we examined the local embedding neighborhoods of bacterial miniproteins relative to metalloproteins and nonmetal proteins. For each miniprotein, we defined a local neighborhood in the embedding space based on its *k* nearest neighbors (Euclidean distance in the t-SNE projection) and computed the fraction of those neighbors belonging to each protein class. This neighborhood composition provides a local measure of embedding similarity, enabling a coarse partitioning of miniproteins into distinct regimes.

The majority of miniproteins are not proximal to either canonical protein class. Approximately 53% of sequences occupy neighborhoods largely devoid of both metalloprotein and nonmetal neighbors, forming a distinct and compact region of embedding space. This compact and partially separated region of the manifold could point to a distinct “miniprotein grammar” that is not captured by the statistical features of larger proteins. As seen in the *E. coli* small proteome, only a small fraction (3–4% of miniproteins) occupy metal-enriched neighborhoods. Instead, most (∼40%) cluster with nonmetal proteins. The remaining ∼3% fall into mixed neighborhoods, reflecting partial overlap between these regimes. Taken together, these observations argue against a simple classification of miniproteins as truncated versions of canonical proteins.

From an evolutionary perspective, this structure is suggestive. A small, metal-associated subset of miniproteins is consistent with models in which metal coordination stabilizes early peptides, while the much larger isolated population raises the possibility that many miniproteins follow alternative organizational principles, perhaps relying on interaction, disorder, or contextual stabilization rather than intrinsic folding. In this view, miniproteins are not merely intermediates between peptides and proteins but may represent a distinct phase of protein sequence space with its own constraints and evolutionary trajectories. This is a preliminary observation, not yet a substantive conclusion, that should motivate deeper investigations into these questions.

If the very first proteins were short peptides, then metal coordination likely played a crucial role in stabilizing structure, facilitating catalysis, and restricting the combinatorial complexity of prebiotically polymerized sequences. Metals may also have allowed the evolution of miniproteins longer than 35 aa from peptides, where symmetry from metal coordination allowed for larger assemblies without extensive hydrophobic cores. Single-chain miniproteins derived from duplication and fusion of peptides could become the building blocks of proteins. The first emergence of hydrophobically stabilized miniproteins is less clear, as peptide intermediates would have lacked sufficient size to create hydrophobic cores, with notable exceptions designed by talented protein engineers. It is possible they may have evolved from metal-binding miniproteins.^87^ Miniproteins persist today, some likely recycled from fragments of larger proteins. Others perhaps belong to a special phase of protein matter that utilizes a sequence grammar distinct from larger globular proteins—driving molecular innovations in modern protein evolution.
